# Effects of sourdough- or regular-bread fermentation, and phytate reduction on iron bioavailability, absorption, and iron status in humans: a systematic review of intervention studies

**DOI:** 10.3389/fnut.2026.1778997

**Published:** 2026-05-05

**Authors:** Anastasios Nikolaou, Ricardo Assunção, Biljana Cvetković, Anthony Fardet, Amparo Gamero, Mónica Gandía, Sandra Mojsova, Birsen Yilmaz, Mary-Liis Kütt, Dushica Santa, Christophe Chassard, Smilja Praćer, Guy Vergères, Sibel Karakaya, Michail Syrpas

**Affiliations:** 1 Department of Molecular Biology and Genetics, Democritus University of Thrace, Alexandroupolis, Greece; 2 Egas Moniz Center for Interdisciplinary Research (CiiEM), Egas Moniz School of Health and Science, Almada, Portugal; 3 Food and Nutrition Department, National Institute of Health Dr. Ricardo Jorge, Lisboa, Portugal; 4 Institute of Food Technology in Novi Sad, University of Novi Sad, Novi Sad, Serbia; 5 INRAE, Unité de Nutrition Humaine (UNH), Université Clermont Auvergne, Clermont-Ferrand, France; 6 Food Technology Area, Preventive Medicine and Public Health, Food Science, Toxicology and Forensic Medicine Department, Faculty of Pharmacy and Food Sciences, University of València, València, Spain; 7 Faculty of Veterinary Medicine, Ss. Cyril and Methodius University, Skopje, North Macedonia; 8 Department of Biological Sciences, Tata Institute of Fundamental Research, Hyderabad, India; 9 ÄIO Tech OÜ, Tallinn, Estonia; 10 Faculty of Agricultural Sciences and Food, Ss. Cyril and Methodius University, Skopje, North Macedonia; 11 Unité Mixte de recherche sur le Fromage, INRAE, UCA, VetAgro Sup, Aurillac, France; 12 Institute for Biological Research Siniša Stanković, National Institute of the Republic of Serbia, University of Belgrade, Belgrade, Serbia; 13 Agroscope, Bern, Switzerland; 14 Department of Food Engineering, Faculty of Engineering, Ege University, Izmir, Türkiye; 15 Department of Food Science and Technology, Kaunas University of Technology, Kaunas, Lithuania

**Keywords:** anaemia, bread, iron absorption, iron bioavailability, phytate, sourdough fermentation

## Abstract

This systematic review, conducted under the COST Action CA20218 “Promoting Innovation of fermented foods” (PIMENTO), aimed to evaluate whether sourdough- and regular-bread fermentation improve iron bioavailability, absorption, and status in humans. Screening of PubMed, Scopus, and Cochrane Library (January 1970–December 2024) identified 8 human intervention studies, in healthy or iron-deficient participants, that met inclusion criteria. EFSA’s scientific guidance for health claim applications, which integrates product characteristics and mechanisms of action to the human studies, was followed, and the extracted data were narratively presented. Results were inconclusive as acute postprandial studies increased non-haem iron bioavailability (especially in low-phytate breads); for example, low-phytate white bread produced a greater 2 h increase in serum iron than high-phytate wholemeal bread (59 vs. 30 μg Fe/100 mL), while exogenous phytase increased iron absorption by 50% for ferrous sulfate and 61% for iron bis-glycine chelate. However, long-term trials did not improve, and in one case even decreased, ferritin and total body iron; specifically, in the low-phytate sourdough rye bread group, ferritin declined from 32 ± 7 to 27 ± 6 μg/L and total body iron from 6.9 ± 1.4 to 5.4 ± 1.1 mg/kg over 12 weeks. On the other hand, phytate reduction combined with iron fortification showed positive effects on haemoglobin or prevented iron depletion; in anaemic children, fermented amaranth bread increased haemoglobin [adjusted *β* = 8.9 g/L (95% CI: 3.5–14.3)] and reduced anaemia prevalence (32% vs. 56%) compared to control bread. Despite convincing mechanistic evidence that the sourdough-fermentation process in bread fabrication improves iron bioavailability, through reduction of phytate, no human studies address this research question with the appropriate control and study quality.

**Systematic review registration:**

osf.io/gzt8m.

## Introduction

Iron Deficiency Anaemia (IDA) is the most common micronutrient deficiency, affecting over 30% of the world population, with childhood and pregnancy being the most indicative periods for its occurrence (1–3). From a broader public health perspective, anaemia not only affects the health status of an individual, but also results in the reduction of work productivity and influences socio-economic development (especially in developing countries) (4–7). In fact, population of low-income countries are the most prone to experiencing IDA, often in synergy with infectious diseases and/or other nutritional deficiencies (like vitamin A, folate, etc) (6, 8).

Iron is known to be an essential mineral for the human metabolism. Howewer, no physiological mechanism for excretion exists, and as a result iron levels in the human body are totally influenced by intestinal iron absorption (9, 10). On this context, iron fortification of food systems remains a widely applied strategy, but its efficacy relies both on the iron form as well as the food matrix involved (9, 11). In a cereal-based diet, iron is mainly present in the non-heme form, and thus, it has to be in a reduced form in order to be transported across the duodenal epithelium. However, several factors may act as enhancers (such as ascorbic acid, folic acid, and others) or as potential inhibitors (such as phytates and polyphenols) (12, 13). For instance, complexation of iron with phytate may impact negatively its absorption (14).

Commonly, bread (or flour) is considered a suitable vehicle for iron fortification, as it is a staple and affordable food product eaten on a daily basis. Importantly, yeast-leavening and sourdough fermentation of bread promotes phytate degradation and may increases iron bioavailability (15–19). Specifically, yeast and lactic acid bacteria metabolic activity lower the pH, enhance phytase activity and lead to the hydrolysis of myo-inositol hexakisphosphate (IP₆), thus reducing its ability to bind minerals (20). This biochemical modification may increase the solubility and availability of the iron fraction aimed for intestinal absorption (21).

Despite indications that the fermentation process and the reduction of phytate levels may enhance the bioavailability of non-heme iron in bread (20), there is a lack of consistent scientific evidence focusing on the role of the mechanism and/or its clinical benefits (22, 23). Different interventions may combine fermentation and iron fortification (without separately evaluating the fortified and the non-fortified sourdough), and often any positive outcomes in iron absorption are not fully justified in the long-term (24, 25). Due to this heterogeneity, the effect of fermentation (and dephytization) on iron absorption remains unclear, and thus, the search strategy used in our systematic narrative review aimed to explore this gap.

This systematic review is part of the COST Action CA20218 Promoting Innovation of ferMENTed fOods (PIMENTO) (26) and aimed to examine the available clinical studies that assessed the association between sourdough fermentation and yeast-leavened bread and iron bioavailability, absorption, and status in human subjects. More specifically, it addresses the question: “Does sourdough- and regular-bread fermentation increase iron bioavailability, absorption, and status in humans?” All types of breads made from cereal flours—with or without prior fortification—will be considered, and iron-related biomarkers such as haemoglobin, haematocrit, serum iron, serum ferritin, serum transferrin receptor, total iron-binding capacity, and iron isotope absorption will serve as key outcomes of interest. This systematic review follows EFSA scientific guidelines, aiming to prepare a structured evaluation of whether the fermentation of bread, with or without iron fortification, meets the criteria necessary to support nutritional claims. The review’s structure adheres to the guidance of the European Food Safety Authority (EFSA) for substantiating health claims and follows a transparent, and reproducible methodology. Ultimately, this work aims to support the development of functional bread products and also to inform public health strategies to mitigate iron deficiency through dietary means.

## Materials and methods

### Systematic review of human studies

This study protocol is one of the systematic reviews conducted by PIMENTO Working Group 3. The systematic review based on this study protocol uses the guidance of EFSA “Scientific and technical guidance for the preparation and presentation of a health claim application” (27), the publication by Muka et al. (28), and the specific EFSA “General scientific guidance for stakeholders on health claim applications” (29). The study reported according to the updated Preferred Reporting Items for Systematic Reviews and Meta-Analyses (PRISMA) (30).

### Literature search

A literature search was conducted to identify human studies investigating the effects of sourdough or regular-yeast bread fermentation, whether or not previously fortified, on iron bioavailability, absorption, and status (Supplementary Table S1).

Different studies (intervention studies, observational studies, cohorts, etc) identified through a systematic search using three electronic databases: PubMed, Scopus, and Cochrane Library, were initially screened between January 1.1970 and August 31. 2023. The literature search was later expanded through December 31. 2024.

### Selection criteria

CADIMA software (31) was used to select the references and eliminate duplicates. Study selection was performed in two stages, according to the steps outlined by Muka et al. (28): in a first stage, the titles and abstracts were screened by at least two reviewers; in a second stage, the full texts were retrieved and the selection criteria applied. CADIMA automatically identified inconsistencies between reviewers, and the reviewers were asked to resolve them. In case of remaining disagreements a third reviewer was advised.

The team researchers used the PICO (population, intervention, comparator, and outcome) criteria to effectively screen documents. The inclusion criteria for the population (P) were as follows: (i) healthy individuals with normal iron status, (ii) individuals with low or marginal iron status, and (iii) iron-deficient or anaemic individuals. Preschool-age children, women of childbearing age, and pregnant women are among the subgroups, which are particularly at risk. Low ferritin levels were interpreted (where applicable) based on the current WHO guidance (32), namely <15 μg/L for adults, and <12 μg/L for preschool children, respectively. The unhealthy population was excluded, except for the iron-deficient individuals, and all animal or *in vitro* studies were also eliminated. Fermented cereals (wheat, oat, or other) used to produce sourdough and yeast-fermented bread, which may or may not have been previously fortified/supplemented, as well as ethnic breads/flatbreads, were considered as intervention (I). Studies with chemically leavened bread were removed, and fermented gruels/porridge were not considered bread. Normal, iron-fortified, or high-phytate bread was used as a comparator (C), depending on each study’s intervention plan. Primary outcomes of interest (O) were iron-related biomarkers, including Haemoglobin (Hb), Hematocrit (Hct), Serum Iron, Ferritin, Serum Transferrin Receptor (sTfR), Total Iron-binding Capacity (TIBC), and Iron Absorption measured with an iron isotope.

### Data extraction

Data extraction was carried out following the guidelines and steps outlined in Muka et al. (28), CADIMA software (31) was used to select references. A consistency test was performed in CADIMA using a subset of the literature dataset. References were selected based on titles, abstracts, and text. Two reviewers extracted data independently. Discrepancies were resolved by discussion or by involving a third reviewer.

Data extraction was completed for human intervention studies that met the PICO criteria, while no observation studies were identified among the final selection. Extracted data were merged and compiled into structured databases.

### Risk of bias assessment

Risk of Bias, for the included PICO studies, was assessed using the Revised Cochrane Risk of Bias tool for Randomized Trials (RoB 2) (33). The RoB 2 has five domains: randomisation process, deviations from intended interventions, missing outcome data, measurement of the outcome, and selection of the reported result. Each domain can be rated as low, some concerns, or high risk of bias. Two reviewers independently completed the RoB step. In the event of any disagreements, the reviewers discussed and reached a final decision. Albeit this, if there were still disagreements between the two reviewers, a third reviewer was involved in the discussions. The results of RoB have been visualised using the RobVis tool (34).

### Study protocol

The methodology follows the framework proposed by Muka et al. (28) and is adapted using the PIMENTO Study Protocol (https://doi.org/10.17605/OSF.IO/GZT8M). The methodological standards of the Cochrane Handbook for Systematic Reviews of Interventions (35) were followed, and the PRISMA 2020 statement (30) was adhered to ensure transparent and comprehensive reporting. The study protocol is registered and publicly available on the Open Science Framework (OSF)^1^ and also published on the PIMENTO webpage.^2^

### Data synthesis

Extracted data from human studies meeting the PICO criteria included: study year, duration, population description, intervention details, phytate context of the meal, key status markers & outcome, and main findings. All primary outcomes were documented as absolute values or as changes from baseline.

Risk of bias in interventional trials (randomisation process, deviations from intended interventions, missing data, outcome measurement, selective reporting, period and carryover effects) was assessed using RoB 2. Methodological or other limitations across studies, which may have led to potential measurement bias, were noted, and knowledge gaps were pinpointed. All data management was conducted in Microsoft Excel, and risk-of-bias visualisations were produced via the Robvis application (33).

### Non-systematic review components

In addition to the formal systematic review of human studies, a narrative (non-systematic) review was performed, as discussed above. Specifically, guided by EFSA recommendations for substantiating health relationships, (a) the characteristics of the fermented foods studied, (b) supportive evidence on iron bioavailability, (c) plausible mechanisms of action and (d) the safety of fermented food consumption were examined.

## Results and discussion

### Literature search

**Figure 1:**
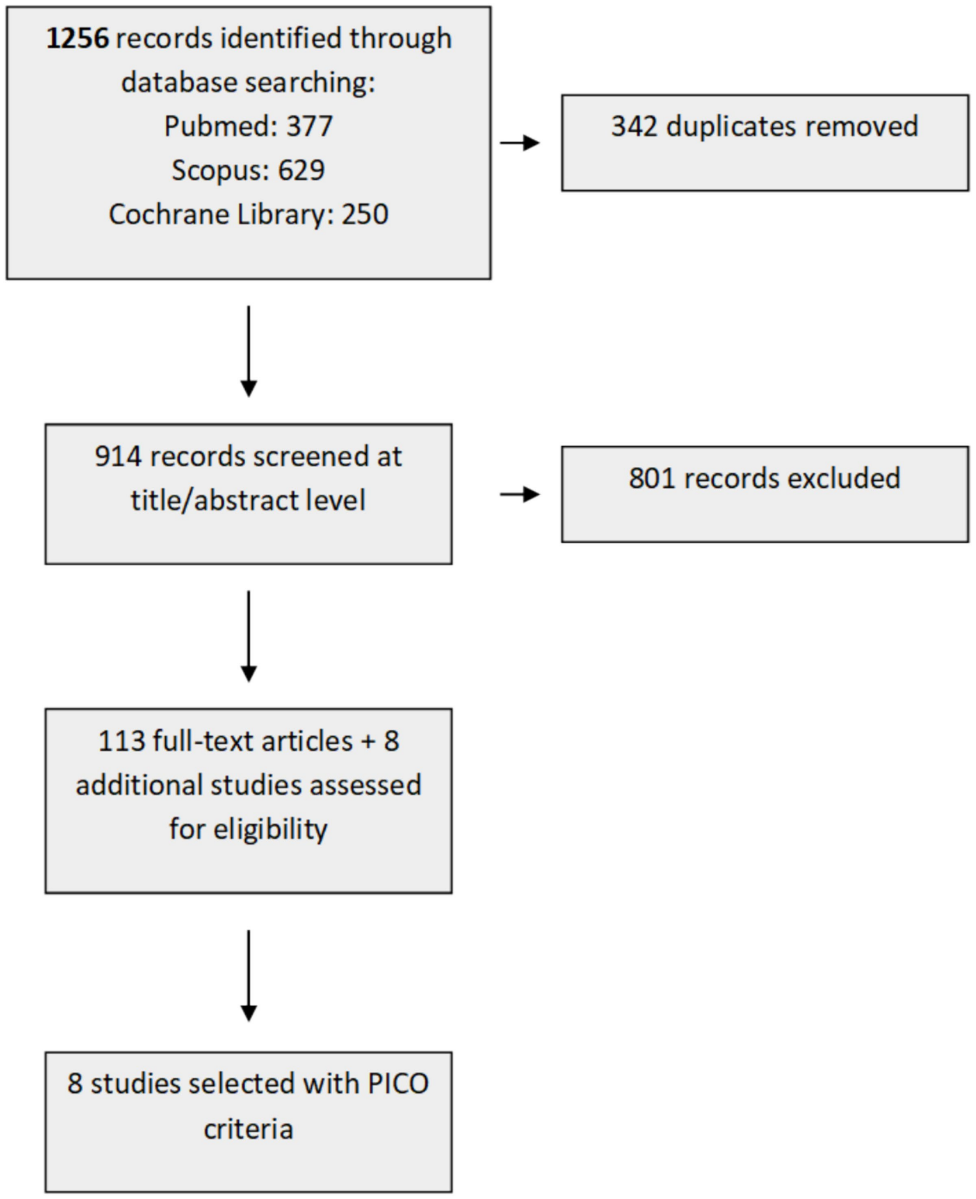
PRISMA flow diagram for the systematic review.

The initial screening identified 1,256 publications across various databases (Figure 1). Duplicates (342) were removed, and after title & abstract screening, 113 studies remained. All studies were directly moved to full-text screening along with 8 additional studies identified from the references of relevant systematic reviews. At this stage, PICO selection criteria yielded 8 studies for the final synthesis.

### Risk of bias assessment in human studies

**Figure 2:**
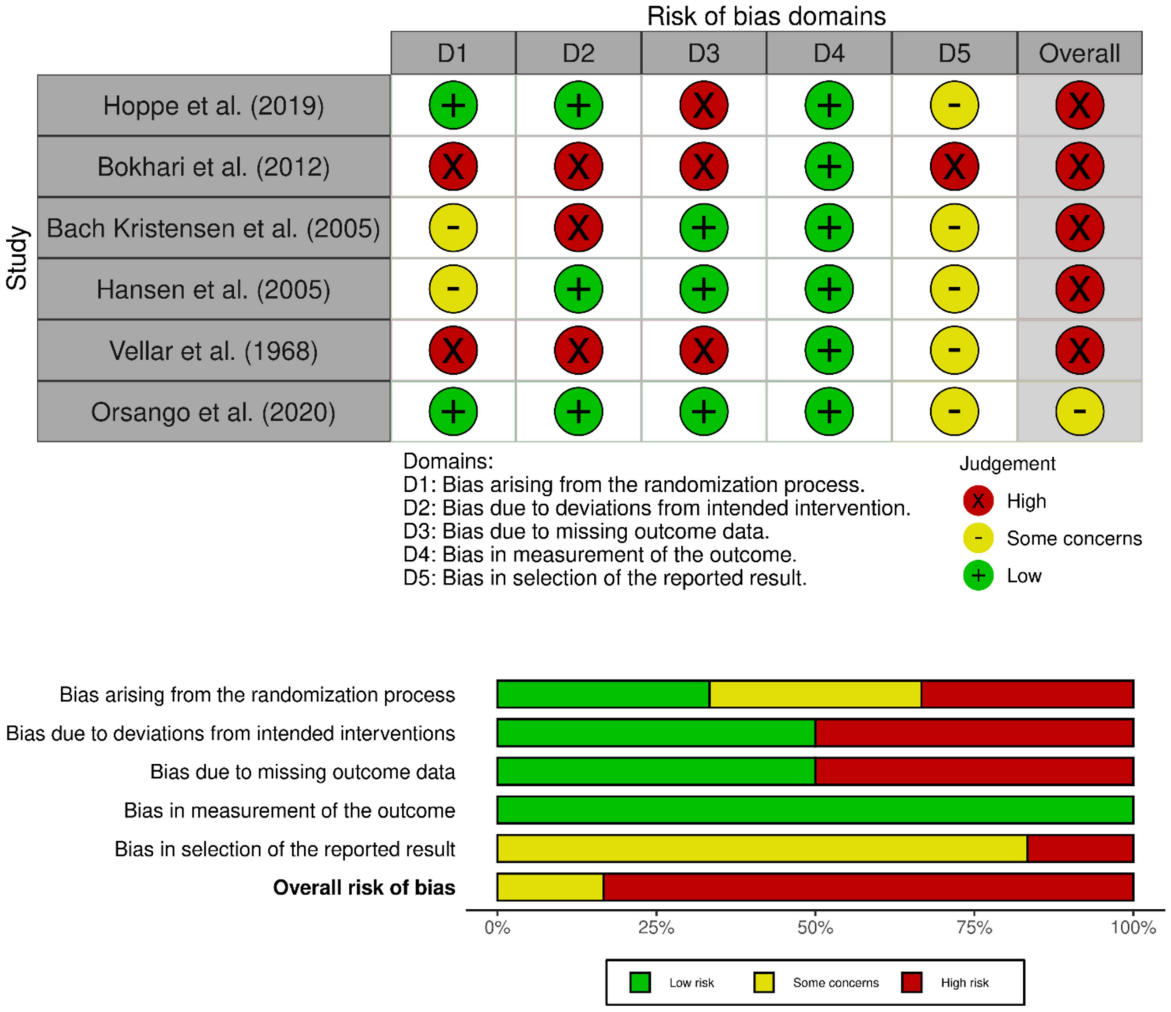
Risk of bias summary of parallel-group trials.

The risk of bias assessment was completed for all 8 studies. Figure 2 summarises the 6 parallel-group trials rated at each risk level across domains. Specifically, five studies were rated as having a high risk of bias (36–40) and one study was judged to have some concerns (41). Overall, the majority of parallel-group trials exhibited high risk of bias, particularly in the domains of deviations from intended interventions (three studies had high risk) and missing outcome data (three studies had high risk). Bias arising from the selection of reported results was either high (36) or raised concerns (37–41) in the included studies. All studies had low risk in terms of measurement of the outcome, suggesting that outcome assessments were conducted consistently and objectively across trials. Taken together, these findings indicate that, while outcome measurement was generally robust, methodological limitations in the interventions, data completeness, and selective reporting may have influenced the reliability of the results from the included parallel-group trials.

**Figure 3:**
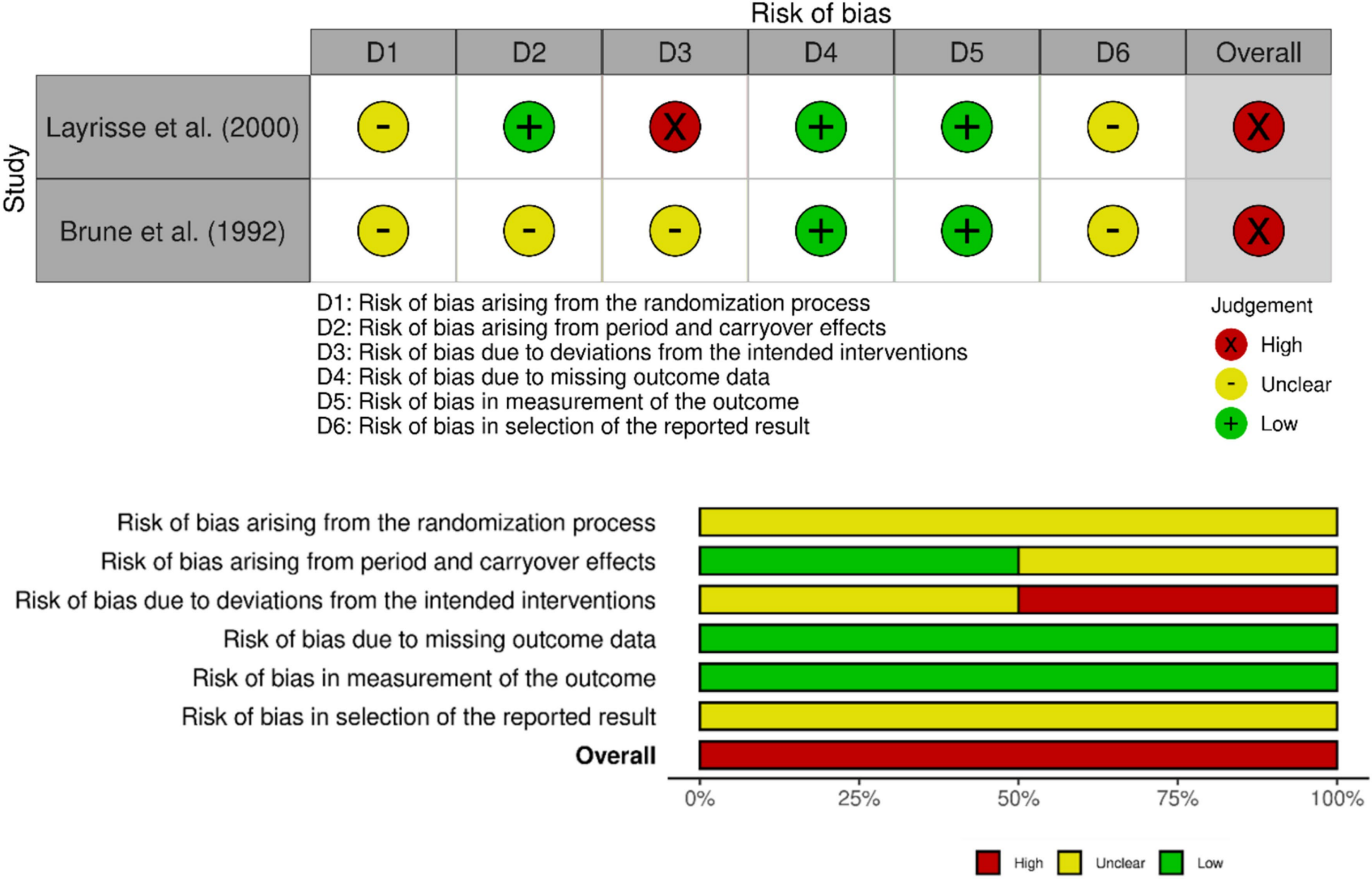
Risk of bias summary of crossover trials.

Figure 3 shows the summary of risk of bias for the two crossover trials, incorporating an additional domain that assesses bias arising from period and carryover effects specific to the crossover design. Both studies (42, 43) had high risk, and the main sources of bias were related to the randomisation process, deviations from intended interventions, and the selection of the reported results. These domains may reflect potential methodological issues, such as inadequate sequence generation, deviations from the assigned intervention across different periods, and selective outcome reporting. Despite these concerns, other domains, such as missing outcome data and outcome measurement, were rated as low risk. Collectively, these findings suggest that while outcome assessments were largely reliable, the internal validity of the crossover trials may have been compromised by procedural and reporting biases inherent to their design. Scientific manuscripts should thus incorporate critical appraisal of study design, including high dropout rates (e.g., 31% in the Hoppe et al. study) and dietary confounders, to better interpret the variability in long-term outcomes.

### Review of human studies on the effects of fermentation on iron bioavailability and status

Past research on the effects of bread fermentation on human iron absorption and status has yielded a complex set of results. The majority of the retrieved studies implicated flour and bread fortification strategies, whose impact on human health has already been comprehensively investigated (44–46). Only a few scientific efforts (37, 42, 47) appear to have explored the effect of fermentation in iron bioavailability, as stated in our research question, although employing very different intervention schemes. The rest of the studies (36, 38–41, 43) approached the scientific question of interest in a rather mechanistic perspective and were associated with processing techniques, primarily aimed at degrading phytate. Therefore, most of the retrieved studies did not examine the inherent biological effect of fermentation itself, but instead implicated mixed intervention schemes in which fermentation, phytate reduction, and/or iron fortification were partially confounded. This limitation is not only quantitative, but also conceptual, since much of the available literature addresses phytate degradation and mineral bioavailability in general, rather than specifically evaluating iron-related outcomes in human interventions with fermented bread (22, 37, 47). Nevertheless, based on the consensus statement on fermented foods and beverages as “foods made through desired microbial growth and enzymatic conversions of food components”, manuscripts focusing on the action of bread fermentation-related enzymes (phytase) were considered as highly relevant to this research’s objectives and included in the final selection. To facilitate the presentation and subsequent discussion, all studies are summarised in Table 1 (acute postprandial studies) and Table 2 (long-term studies). Moreover, the merged detailed data extraction tables are available in Supplementary Table S2.

**Table 1:** Acute postprandial bioavailability studies.

Study (Year)	Population description	Intervention vs comparator	Characterization of intervention	Phytate context of meal	Primary absorption outcome	Main findings and context
Brune et al. (42)	9–10 healthy subjects (M/F) per experiment.	Sourdough-fermented whole rye+wheat rolls. Contol wheat rolls: with very low content of inositol phosphate.	Prolonged sourdough fermentation at 23 °C for 48 h, before the final dough preparation. Yeast was used in all breads. Residual IP was quantified by HPLC. No data reported for: pH, TTA, LAB strains, and phytase activity.	Prolonged (48h) sourdough fermentation reduced inositol phosphates (ΣIP_3–6_) to very low levels (similar to the controls).	Fractional absorption ratio was 0.94 ± 0.05 (not significantly different from the controls).	Effective fermentation significantly improved iron bioavailability; inhibition was associated with residual inositol phosphates (IP_3_-IP_6_).
Studies focusing on the mechanistic effect of phytate reduction						
Vellar et al. (40) (Absorption experiment)	20 young, healthy female volunteers.	Bread fortification with ferrous sulphate (∼30 mg Fe/100 g).	Wholemeal bread was made from equal parts of 78 and 100% extraction wheat flour. White bread was made from 78% extraction wheat flour. No data reported for: fermentation parameters, starter cultures, pH, TTA, phytase activity, residual inositol phosphate profile, or phytate:iron molar ratio.	HP wholemeal bread: 71 mg phytic acid phosphorus/100 g. LP normal white bread: 29 mg phytic acid phosphorus/100 g.	Mean increase in serum iron (2h post-meal): 59 μg Fe per 100 mL (LP) vs. 30 μg Fe per 100 mL (HP).	The inhibitory effect of dietary phytate in wholemeal bread was only partial, as a marked increase in serum iron was still observed.
Layrisse et al. (43)	14 subjects (M/F).	Exogenous phytase addition (304 U) to fortified bread (with ferrous sulfate or ferrochel).	No data reported for: fermentation parameters, starter cultures, pH, TTA, residual inositol phosphate profile, or phytate:iron molar ratio.	Phytate-rich corn flour (mean 168 mg/100g).	Iron absorption increased by 50% for ferrous sulfate and 61% for ferrochel in the presence of phytase (*p* < 0.05 for both).	Phytase addition significantly increases the bioavailability of both ferrous sulfate and ferrochel by degrading phytate. Ferrochel partially prevents phytate inhibition.
Bokhari et al. (36)	24 non-pregnant, premenopausal women (pilot trial).	Exogenous phytase addition (Level 2: 0.015 g/100 g flour) to Teff bread (TB).	Teff bread (TB) was made using 50% of teff flour. No data reported for: fermentation parameters, baking, pH, TTA, phytase activity and phytate content, residual inositol phosphates, or phytate:iron molar ratio.	TB was iron-rich (4.1 mg Fe/100 g), but contained inhibitors.	Smallest mean reduction in serum iron: −0.3 μm (TB + P2) vs. −1.5 μm (average of all bread groups). Highest AUC.	Phytase improved iron absorption, but intrinsic iron levels alone were insufficient to prevent overall serum iron decline in fasted subjects.

M, Male; F, Female; HP, High-phytate; LP, Low-phytate; TB, Teff bread; TTA, Total Titratable Acidity; AUC, Area Under the Curve.

**Table 2:** Long-term bioavailability studies.

Study (Year)	Duration	Population status (Baseline SF)	Intervention vs comparator	Characterization of intervention	Key status markers	Main findings and context
Hoppe et al. (37) and Hoppe et al. (47)	3 months	Healthy Swedish females (SF: 31–33 μg/L).	Sourdough-fermented LP bread: <1.0 mg of phytate/200 g. Normal HP bread: 77 mg of phytate/200 g.	10% sourdough added to dough. Sourdough fermentation for 24 h. Baking at 200 °C for 55 min. LAB strains not reported. pH 4.0 (for low-phytate) vs. 4.6 (for high-phytate). Organic acids were measured. No data reported for: TTA and phytase activity.	Sourdough-fermented LP group: SF decreased significantly (mean 12%, from 33 → 27 μg/L, *p* < 0.018). Total body Fe decreased significantly (mean 12%, *p* < 0.035). Normal HP group: No changes in iron biomarkers.	The LP strategy alone failed and unexpectedly reduced iron biomarkers. This outcome was linked to confounding dietary changes, specifically a decrease in the consumption of iron enhancers such as meat, fish, and/or poultry.
Studies focusing on the mechanistic effect of phytate reduction						
Bach Kristensen et al. (38)	4 months	Healthy young women; adequate iron levels (SF: 45 μg/L).	Daily consumption of 300 g fibre-rich wheat bread. Phytase addition only achieved a 23% reduction in ΣIP_3–6_ and 18% in ΣIP_5–6_, respectively. Phytate:iron molar ratio was 8.5:1 (for HP) and 6.7:1 (for LP).	Conventional yeast-leavening. *Aspergillus phytase* used (2500 PTU/100 g). Dough preparation: 73 min (6 min mixing, 12 min at 22 °C, 55 min at 36 °C). Baking at 220 °C for 32 min. Bread pH 5.6. TTA not reported.	SF: Decreased significantly by 27% (45 → 33 μg/L). Hb: Decreased significantly by 1.5%.	Consumption of the recommended daily intake of fibre-rich bread impaired iron status. The reduction in phytate (23% for ΣIP_3–6_ and 18% for ΣIP_5–6_), was insufficient to maintain iron status.
Hansen et al. (39)	5 months	Young marginally iron-deficient women (SF: 19.2–24.6 μg/L).	Sourdough rye bread (LP, ΣIP_5–6_ ≈ 70 mmol/100 g). Fortified with ferrous fumarate (8.6 mg Fe/day).	Sourdough contributed 20–30% of the flour and was fermented for 10–14 h. Final dough process: 10 min mixing, 20 min rest, 70 min proof, and 70 min baking at 220 → 180 °C. Endogenous phytase allowed to act for ~135 min. No data reported for: dough pH, TTA, LAB strains, or phytase activity.	SF (fortified group): 19.2 → 18.3 mg/L. No change (*p* = 0.85). SF (unfortified group): 24.6 → 20.2 mg/L. Significantly reduced (*p* = 0.02).	Fortified, LP bread stabilised iron levels, counteracting the decrease observed in the control group consuming unfortified bread.
Orsango et al. (41)	6 months	Anaemic children (Hb < 110.0 g/L).	Amaranth bread (70% Amaranth +30% Chickpea) processed via soaking, germinating, and fermenting (to decrease phytate).	Initial amaranth grain processing: soaking for 24 h in acidified water (5 mL lemon juice/100 mL water), germinating for 72 h, sun drying, roasting, and milling. Nutrient content of breads reported. No data reported for: fermentation parameters, starter cultures, pH, TTA, phytase activity, phytate content of the final breads, residual inositol phosphate profile, or phytate:iron molar ratio.	Hb: The amaranth group had a significantly greater increase (adjusted β = 8.9 g/L, *p* < 0.01). Anemia prevalence: Significantly lower in the Amaranth group (32%) vs. in the Maize group (56%); aRR: 0.39.	Processed amaranth bread had favourable effects on Hb concentration and reduced anaemia prevalence, attributed to phytate reduction, which enhanced non-heme iron absorption.

SF, Serum Ferritin; HP, High-phytate; LP, Low-phytate; Hb, Haemoglobin; TTA, Total Titratable Acidity.

Initially, in acute postprandial absorption tests, the study of Vellar et al. (40) demonstrated the inhibitory effect of wholemeal (high-phytate) bread (containing 71 mg phytic acid phosphorus per 100 g) that resulted in decreased serum iron concentrations (2 h after ingestion) in 20 young healthy women, compared to the normal-phytate white bread (29 mg/100 g) (Table 1). In the same context, Brune et al. (42) suggested that the inhibition of iron absorption by wheat and rye was attributed to their high phytate content, as well as to their degradation products (IP_3_-IP_6_). By employing radio-iron tracers, the authors found that prolonged sourdough fermentation reduced phytate (and total inositol phosphates) to levels similar to those in the control white rolls. As a direct effect, the iron bioavailability of sourdough-fermented whole-meal bread was improved.

The intentional phytate reduction (in single-meal settings) and its effects on iron bioavailability was further explored by studies focusing in flour fermentation with the addition of phytase (Table 1). Specifically, in the study of Layrisse et al. (43), authors showed that when 304 U phytase was added to a phytate-rich corn flour breakfast, iron absorption increased significantly by 50% for ferrous sulfate and 61% for iron bis-glycine chelate (ferrochel) (*p* < 0.05), compared to the respective basal breakfast without phytase. Notably, the addition of phytase doubled the absorption of both compounds. Similarly, Bokhari et al. (36) showed that the addition of phytase to iron-rich Teff bread consumed by non-pregnant women resulted in the least reduction in serum iron (e.g., in the TB + P2 group, the reduction was −0.3 μm) and the highest indicator of absorption (Area Under the Curve analysis) in comparison to plain Teff bread. However, it should be noted that any improvement observed was only relative, as the serum iron levels still declined in all groups consuming bread interventions, thus reflecting the complexity of non-heme iron absorption.

Respectively, Hoppe et al. (37) and Hoppe et al. (47) was the only long-term intervention study to test sourdough-fermented wholegrain rye bread (over 3 months) for the potential improvement of iron status in healthy Swedish women. Interestingly, sourdough fermentation resulted in significant phytate reduction (to <1.0 mg phytate/200 g bread) compared to the normal (high-phytate) rye bread (77 mg/200 g). The rye (high-phytate) bread group (*n* = 31) showed no change in any iron status biomarkers, but surprisingly the sourdough (low-phytate) bread group (*n* = 24) experienced significant within-group decreases in both ferritin (mean 12%; from 32 ± 7 to 27 ± 6 μg/L) and total body iron (mean 12%; from 6.9 ± 1.4 to 5.4 ± 1.1 mg/kg). Furthermore, there was a significant difference in the change over time in total body iron levels between the two groups (*p* < 0.035). Researchers assumed that this unexpected reduction may be explained by confounding dietary changes in the sourdough (low-phytate) group, including documented significant decreases in the frequency of coffee and tea intake (*p* = 0.003) and in meat, fish, and/or poultry consumption (*p* = 0.017), along with a tendency to decrease vitamin C intake (*p* = 0.053). The study’s high dropout rate (31%) and its free-living design, which limited control over dietary intake, were also noted as key limitations.

In any case, the limited consumption of iron enhancers should not be considered exclusively responsible for the documented decreases, as other mechanistic pathways might also be implicated. For instance, the low pH (due to sourdough fermentation) is normally expected to have a positive impact by enhancing the phytase activity and the subsequent phytate degradation (48). However, the reduction of IP6 may not be effective, since the presence of residual inositol phosphates is also capable of limiting iron absorption (49, 50). Most importantly, improved iron bioavailability after bread consumption (in a single meal) does not necessarily ensure high body iron stores in the long-term. As concluded in a recent crossover study (published after the end of the review period), by the same authors (25), despite the clear advantages of sourdough-derived dephytization, the nutritional relevance still remains constrained by aspects like the low initial iron content of the food matrix (bread), the subject’s diet, the adherence to meals, and the human physiological regulation.

The positive effect of long-term (low-phytate) bread interventions was also demonstrated for iron status biomarkers (serum ferritin, haemoglobin, total body iron) in vulnerable populations or when fortification strategies were included (Table 2). For instance, Hansen et al. (39), in a 5 month study of marginally iron-deficient women, used sourdough-fermented rye bread, with relatively low phytate content (∼70 μmol/100 g ΣIP_5-6_), fortified with ferrous fumarate. While this bread did not increase iron status (serum feritin remained unchanged), it successfully prevented the significant decline in serum ferritin observed in the unfortified control group (*p* = 0.02), thereby stabilising iron levels. Furthermore, Orsango et al. (41) demonstrated efficacy when combining multiple processes (soaking, germination, and fermentation) in amaranth bread consumed by anaemic children. After 6 months, the amaranth bread group showed a significantly higher increase in mean haemoglobin concentration [adjusted *β* = 8.9 g/L (95% CI: 3.5–14.3)] and a significantly lower anaemia prevalence (32% vs. 56%) compared to the maise bread control group (*p* < 0.01 for both). This success was attributed to the processing steps, which reduced phytate levels and enhanced iron absorption in the micronutrient-rich amaranth grain.

On the other hand, unexpected results, highly inconsistent with the low-phytate mechanism hypothesis, were reported in the study by Bach Kristensen et al. (38). Specifically, the consumption of 300 g/day of fibre-rich wheat bread was tested over 4 months in healthy young women with initially sufficient iron levels. Phytase addition (2500 U/100 g) achieved only a modest reduction in phytate (23% for ΣIP_3–6_ and 18% for ΣIP_5–6_), which was insufficient to improve iron bioavailability, as the residual phytic acid:iron molar ratio remained high (6.7:1 in the phytase-added bread vs. 8.5:1 in the normal control bread). As a result, no beneficial comparative effect of phytase addition on iron status was observed and, on the contrary, the consumption of the bread resulted in a general impairment of iron status: Serum ferritin decreased significantly by 27% (e.g., from 45 μg/L to 33 μg/L) and haemoglobin decreased by 1.5% (2 ± 0.8 g/L) after 4 months.

Overall, indications from acute postprandial trials show that effective sourdough fermentation significantly increases iron bioavailability (by degrading phytates) (42). The same outcome may be mechanistically achieved by adding phytase to bread. Although determination of phytate content was reported in some studies (by applying AOAC and HPLC methods), a direct comparison of the absolute phytate reduction was feasible only in specific subsets of trials, as several studies lacked bread phytate data, appropriate comparators, or sufficient reporting for calculation (37, 42, 47). As a result, there was not enough available evidence to support an optimal phytate:iron molar ratio threshold that would indicate improvement of iron status. Nevertheless, successful improvement or stabilisation of iron status appears more likely when the phytate reduction is maximised and applied to iron-rich foods or fortified vehicles (as seen above with amaranth and fortified rye bread), especially when targeting populations with existing low iron reserves or when favourable dietary enhancers are maintained. It should be noted though that the overall effect on long-term iron status remains highly context-dependent (37, 47), as the observed outcomes are highly variable and often framed within methodological limitations, such as high dropout rates, uneven subjects’ habitual diets, and others.

One key limitation of the present synthesis is the relatively high risk of bias across the included studies, as reported in the previous sections of the paper. Most of the studies were rated as having a high overall risk of bias, especially in the domains of deviations from intended interventions, missing outcome data, and selection of reported results. These methodological concerns may impact the confidence that can be placed in the observed findings and limit the strength of the narrative synthesis. The bias related to selective outcome reporting may have influenced the apparent pattern of results across studies. Acute postprandial trials frequently reported increases in iron bioavailability or absorption, whereas longer-term trials evaluating iron status markers often reported null or inconsistent effects. It is possible that positive outcomes in short-term trials are more likely to be reported or emphasized, while null findings from longer interventions may be underreported. Therefore, the differences between acute bioavailability outcomes and longer-term iron status indicators should be interpreted cautiously. As a conclusion, the available human evidence remains limited and methodologically heterogeneous, highlighting the need for well-designed randomized controlled trials with standardized outcomes and adequate follow-up duration.

### Effects of sourdough- and regular-bread fermentation on iron bioavailability in *in vitro*, and *in vivo* studies

Several studies have reported the beneficial impact of fermentation (especially sourdough) on the bioaccessibility of minerals, such as iron, calcium, magnesium, and zinc. For example, an *in vitro* digestibility of +9–24% has been calculated for the total mineral content of sourdough breads with either starter (phytase-active LAB strain) or spontaneous fermentation, the effect being mainly attributed to phytic acid degradation by phytate-degrading enzymes (51). Concerning endogenous iron more specifically, it’s *in vitro* release (i.e., bioaccessibility) may be increased by 8-fold in sourdough bread compared to conventional bread (52). However, this does not always translate to higher bioavailable iron levels through the metabolic pathway. As demonstrated, in vivo, other unknown factors such as the food matrix effect or human physiological regulation by hepcidin release (25, 37, 47), may affect and regulate the iron absorption depending on the needs of human organism (53). Such observations indicate the complex interactions among dietary patterns, suggesting that other food habits, like the number of coffee and tea servings and/or the frequency of meat intake accompanying low-phytate bread interventions, may impact thereafter the real iron bioavailability, in real-life conditions (54).

In another study, it was estimated that a fermentation temperature of 25 °C might be optimal for some lactic acid bacteria (LAB) strains and, subsequently, for the endogenous mineral content of sourdough breads, resulting in significantly higher phytase activity (55). An increased iron content of 36–45% was also reported for traditional Iranian sourdough breads, suggesting a subsequent increase in bioaccessibility, which may lead to higher bioavailability (56). Otherwise, in an *in vitro* Caco-2/HepG2 (human liver cells) cell model, sourdough bread was associated with a 53% increase in ferritin formation and a 97% increase in hepcidin release compared with non-sourdough heat-treated bread (57). Besides, improved *in vitro* iron bioavailability in sourdough bread was confirmed in rats, showing a + 20% increase in apparent iron absorption (58), and in mice receiving quinoa snacks elaborated from sourdough-fermented with a phytase-positive strain (59).

### Mechanistic basis of phytate–iron interactions

Iron deficiency anaemia remains one of the most prevalent nutritional disorders globally, and cereal-based diets are a significant contributing factor due to their high phytic acid (myo-inositol hexakisphosphate, IP₆) content. Phytate serves as the principal storage form of phosphorus in cereals and legumes, but its strong chelating capacity toward divalent and trivalent cations (Fe^2+^/Fe^3+^, Zn^2+^, Ca^2+^, Mg^2+^) significantly limits mineral absorption (60). At intestinal pH, deprotonated phosphate groups in phytate form insoluble complexes with ferric iron, rendering it unavailable for absorption (9). This effect is particularly pronounced for non-haem iron, the predominant form in cereals, which has a variable absorption rate of 1–22%, compared with 15–35% for haem iron (61). The inhibitory effect of phytate depends on its absolute concentration, molar ratios to iron, and the specific distribution of iron between ferritin and phytate-bound pools, which in turn are influenced by crop genotype, soil conditions, and post-harvest processing (62).

Mechanistically, phytase enzymes (EC 3.1.3.8 and EC 3.1.3.26) play a pivotal role in reducing phytate’s mineral-binding capacity. These enzymes, present endogenously in cereals and exogenously in microorganisms such as lactic acid bacteria (LAB) and yeasts, catalyse the stepwise hydrolysis of phytate into lower inositol phosphates and inorganic phosphate (63). Each successive hydrolysis step diminishes phytate’s chelating capacity, ultimately releasing iron in soluble forms more amenable to intestinal uptake (50, 64). The process is highly dependent on environmental conditions (particularly pH, temperature, and moisture), which align optimally during bread fermentation. Collectively, current evidence indicates that the reduction of phytate and the concomitant enhancement of iron bioavailability during sourdough and bread fermentation result from a multifactorial process in which phytase-mediated hydrolysis, acidification, microbial activity, and processing conditions such as proofing and baking act in concert to diminish phytate levels and increase the fraction of bioavailable iron (48, 65–67).

### Mechanistic pathways in fermentation, breadmaking and iron bioavailability

During every breadmaking process, endogenous cereal phytases and microbial phytases from yeasts and LAB act together to degrade the present phytic acid. The fermentation process lowers the dough pH to around 4–5, which creates an acidic environment optimal for phytase activity, subsequently stimulating phytate hydrolysis (66). LAB also produce lactic and acetic acids, which enhance phytase activity and stabilise iron in soluble complexes, protecting it from re-precipitation (68, 69). Finally, the fermentation-induced breakdown of cell wall polysaccharides and protein–mineral complexes releases the usually bound minerals, further increasing their solubility (67).

Studies from previous decades show that fermentation significantly reduces phytate levels in bread. Quantitatively, reported reductions ranged from 40% to over 90%, depending on fermentation time, microbial strains, and flour extraction rate (64, 70, 71).

Several authors pinpoint that sourdough fermentation is very effective due to the influence of phytase-producing LAB. However, yeast-based and mixed fermentations also contribute to phytate degradation (48, 72). The reduction in phytate content is not limited only to sourdough bread, conventional yeast breads also exhibit this process on a smaller scale. This phenomenon is more intensive when fungal phytase is supplemented (73).

The baking step also contributes to phytate reduction. Heat-induced structural changes to the cereal matrix, along with the residual phytase activity, synergistically promote additional phytate hydrolysis and mineral release (74). Breads produced with prolonged fermentation and baking showed reduced phytate content and substantially improved iron solubility and bioaccessibility in *in vitro* digestion models (75, 76).

Research evidence from Caco-2 cells and human subjects indicates that bread with reduced phytate content enhances iron absorption. This improvement in iron absorption can potentially increase iron levels in individuals (9, 72).

### Bread characterisation

Different kinds of flour (in varying proportions), salt, and water are the basic ingredients for breadmaking. Other additions (such as milk, oil, herbs, sweeteners, fruits, etc.) may contribute to the sensory characteristics (flavour, softness, appearance) and shelf life of the bread (77). The addition of sourdough or baker’s yeast (*Saccharomyces cerevisiae*) is responsible for leavening during fermentation by consuming the sugars present in dough and generating carbon dioxide and ethanol (78). Scientific evidence indicates that the metabolic activity of certain bread-making microorganisms serves other functions as well (79) and critically influences the product’s technological performance, nutritional properties, and overall quality (23, 80).

Out of the 8 papers of the final selection, both studies that used sourdough-fermented bread, in acute postprandial (42) or long-term interventions (37), applied AOAC and/or chromatography methods for determining phytate content and its degradation products. Brune et al. (42) additionally provided measurements for all different flour mixtures they examined, and Hoppe et al. (37) provided the iron content (2.4–2.5 mg) and the organic acids profile of the bread. Similarly, the rest of the studies (focusing on the mechanistic effect of phytate reduction) determined the concentration of phytate, inositol phosphates, iron, and/or calcium in the produced breads by HPLC methods (38, 39) or other chemical analysis protocols (40, 43). Orsango et al. (41), also expanded to the nutrient content (protein, fat, energy) of the amaranth and maize bread they produced, while Bokhari et al. (36) stated that the teff flour they used contained 4.1 mg of iron per 100 g (wet basis). In all cases, ingredient lists and detailed instructions on manufacture and baking were provided.

### Product safety

Regarding the safety of bread as a food matrix for iron bioavailability studies, it is important to note that bread is produced by baking at high temperatures, which, combined with its low water activity, significantly reduces the microbiological risk associated with this product (81, 82). Nonetheless, as a cereal-based product, bread may be susceptible to chemical contaminants, such as mycotoxins, which can originate from fungal contamination of raw materials, especially mouldy grains (83–85). Moreover, acrylamide formation during baking represents an additional chemical hazard, as this compound is generated via Maillard reactions at high temperatures and can consequently contaminate bread samples (86, 87). Despite these potential safety concerns, the existing literature on the bioavailability of iron in bread consistently reports no adverse effects from microbial or chemical hazards.

Although these contaminants are recognised chemical hazards in cereal-based foods, there is currently no consistent evidence demonstrating that their presence directly interferes with iron chelation mechanisms or intestinal iron transport in humans. However, fermentation, particularly sourdough fermentation involving lactic acid bacteria (LAB) and yeasts, may contribute to improving the overall safety profile of bread, especially in the context of mycotoxins contaminantion. Several studies have shown that selected LAB strains are capable of reducing mycotoxin levels through mechanisms such as binding to bacterial cell walls or biotransformation into less toxic metabolites (88–90). In cereal-based matrices, fermentation has also been reported to decrease the concentration of certain Fusarium toxins, including deoxynivalenol, although the extent of reduction depends on the microbial diversity and process conditions (85). Therefore, while food contaminants are not known to directly affect iron bioavailability, fermentation processes, especially those driven by LAB, may enhance the safety of bread as a food matrix for nutritional interventions by mitigating relevant chemical hazards.

## Conclusion

This review aimed to systematically assess whether sourdough- or yeast-leavened bread, and phytate reduction increases iron bioavailability, absorption, and status in humans. However, the results remain rather contradictory. Specifically, postprandial absorption experiments demonstrated that sourdough-leavening of bread enhances non-heme iron bioavailability by promoting phytate degradation during fermentation, a process that mainly relies on microbial activity, acidification, and organic acids production. Iron bioavailability was also shown to be improved when added phytase was used, but this should probably be regarded as a completely different approach rather than as an equivalent model to sourdough fermentation. On the other hand, long-term human intervention trials indicate that these improvements do not necessarily favor iron status under free-living conditions and may even lead to reductions in serum ferritin and total body iron. Importantly, the more promising results (in the long-term) were mainly observed when phytate reduction was combined with iron fortification protocols or with iron-rich vehicles.

In conclusion, findings should be carefully interpreted, as their clinical effectiveness is mostly context-dependent and critically influenced by the study design, the subjects’ overall health, diet quality, and even habitual lifestyle. Future approaches should aim to decipher the low-phytate hypothesis with high-quality trials of adequate duration and appropriate controls, while clearly distinguishing between sourdough-driven and phytase-driven interventions. Important parameters like fermentation conditions, dough pH, total titratable acidity, organic acid profile, phytase source and activity, residual inositol phosphate profile, phytate:iron molar ratio, dietary control, and adherence should also be properly measured and critically examined.

## Data Availability

The original contributions presented in the study are included in the article/Supplementary material, further inquiries can be directed to the corresponding author/s.

## Footnotes

1
https://osf.io/gzt8m/

2
https://fermentedfoods.eu/
